# Symptomatic Atherosclerotic Disease and Decreased Risk of Cancer-Specific Mortality: A Prospective, Population-Based Study (NEDICES)

**DOI:** 10.1097/MD.0000000000001287

**Published:** 2015-08-14

**Authors:** Julián Benito-León, Jesús González de la Aleja, Antonio Martínez-Salio, Elan D. Louis, Judith H. Lichtman, Félix Bermejo-Pareja

**Affiliations:** From the Department of Neurology, University Hospital “12 de Octubre” (JB-L, JGdlA, AM-S, FB-P); Department of Medicine, Faculty of Medicine, Complutense University (JB-L, JGdlA, AM-S, FB-P); Centro de Investigación Biomédica en Red sobre Enfermedades Neurodegenerativas (CIBERNED), Madrid, Spain (JB-L, FB-P); Department of Neurology, Yale School of Medicine (EDL); Department of Chronic Disease Epidemiology, Yale School of Public Health (EDL, JHL); and Center for Neuroepidemiology and Clinical Neurological Research, Yale School of Medicine and Yale School of Public Health, New Haven, CT, USA (EDL, JHL).

## Abstract

The few studies that have assessed the association between symptomatic atherosclerotic disease and risk of cancer have had conflicting results. In addition, these studies ascertained participants either from treatment settings (ie, service-based studies) or by using a records linkage system (ie, medical records of patients evaluated at clinics or hospitals) and, therefore, were prone to selection bias. Our purpose was to estimate the risk of cancer mortality in a large population-based sample of elderly people, comparing participants with symptomatic atherosclerotic disease (atherosclerotic stroke and coronary disease) to their counterparts without symptomatic atherosclerotic disease (ie, controls) in the same population.

In this population-based, prospective study (Neurological Disorders of Central Spain, NEDICES), 5262 elderly community-dwelling participants with and without symptomatic atherosclerotic disease were identified and followed for a median of 12.1 years, after which the death certificates of those who died were reviewed.

A total of 2701 (53.3%) of 5262 participants died, including 314 (68.6%) of 458 participants with symptomatic atherosclerotic disease and 2387 (49.7%) of 4804 controls. Cancer mortality was reported significantly less often in those with symptomatic atherosclerotic disease (15.6%) than in controls (25.6%) (*P* < 0.001). In an unadjusted Cox model, risk of cancer-specific mortality was decreased in participants with symptomatic atherosclerotic disease (HR = 0.74, 95% confidence interval [CI], 0.55−0.98, P = 0.04) vs. those without symptomatic atherosclerotic disease (reference group). In an adjusted Cox model, HR = 0.58; 95% CI, 0.38−0.89; *P* = 0.01.

This population-based, prospective study suggests that there is an inverse association between symptomatic atherosclerotic disease and risk of cancer mortality.

## INTRODUCTION

The worldwide burden of cancer and atherosclerotic disease (ie, atherosclerotic stroke and coronary disease) is expanding rapidly as a result of ageing and/or unhealthy lifestyle.^1–3^ The International Agency for Research on Cancer estimated that in 2012, there were 14.1 million new cases of cancer and 8.2 million deaths.^1^ In 2010, there were approximately 11,569,000 incident ischemic stroke events worldwide,^2^ while for coronary disease, in 2001, there were 7.3 million deaths and 58 million disability adjusted life years lost worldwide.^3^ Common risk factors, including tobacco use, exposures to ionizing radiation, arsenic, and various industrial combustion effluents containing polycyclic aromatic hydrocarbons, sex hormones, unhealthy lifestyle, and being overweight, might favor simultaneous occurrence of both diseases.^4^ In addition, a series of shared molecular pathways play a significant role in the pathogenesis and progression of both atherosclerosis and cancer; these include major molecular inflammatory pathways and angiogenesis, among many others.^5,6^ As a result, the probability of co-occurrence in the same patient of 2 of the most common age-associated diseases worldwide is expected to rise with increasing age.

Based on these considerations, several studies have assessed the incidence of cerebrovascular or cardiovascular disease among patients with active cancer.^7–11^ Overall, these studies point to an increased incidence of cerebrovascular disease among patients with active cancer, mainly during the first months after diagnosis of cancer and then slowly decreasing overtime, which suggests that the association of active cancer and subsequent stroke is mediated by cancer-related mechanisms such as the hypercoagulability state, cancer itself, or the effects of treatment.^8,11^ On the contrary, fewer studies have examined the incidence of cancer among those who suffer from cerebrovascular or cardiovascular disease, and they have reported conflicting results.^12–16^ All studies enrolled subjects from treatment settings (ie, service-based) or used a records linkage system (ie, medical records of patients evaluated at clinics or hospitals) and therefore, were prone to selection bias.^7–16^ Further, most were not adjusted for smoking, an important confounder, since this unhealthy habit increases the risk of both atherosclerotic disease and cancer.^7,9–13,15,16^ In addition, these studies did not differentiate between the main causes of stroke (ie, atherosclerotic strokes vs cardioembolic strokes).^7–16^ The absence of such differentiation could attenuate the ability to detect a relationship between stroke (esp., atherosclerotic disease) and cancer.

To date, it remains unclear whether atherosclerotic disease is associated with an increased risk of cancer. Population-based studies are preferable for studying this relationship because they minimize potential sources of bias and confounding in comparison to those based on hospital discharge registers, and are also preferential when investigating risk factors associated with disease incidence and mortality.^17,18^ Surprizingly, no study has assessed long-term cancer-mortality risk in a population-based cohort of subjects documented to have atherosclerotic cerebrovascular disease or coronary vascular disease.

There is considerable evidence from epidemiological and clinical studies that shows a consistently lower than expected occurrence of cancer in patients with Parkinson disease, Alzheimer disease, multiple sclerosis, and Huntington disease.^19^ We hypothesized that the cancer-mortality risk in participants with symptomatic atherosclerotic disease would be lower than in participants without atherosclerotic disease (ie, controls). To address this question, we utilized data from the Neurological Disorders in Central Spain (NEDICES) study, in which a population-based sample of participants was prospectively evaluated, thus permitting the minimization of selection bias.^20–32^ Our analyses adjusted for numerous potential confounders. In addition, we conducted secondary analyses stratified by smoking status and differentiated between the main causes of stroke (ie, atherosclerotic strokes vs cardioembolic strokes).

## MATERIAL AND METHODS

### Study Population

Data for these analyses were derived from the NEDICES study, a longitudinal, population-based survey of the prevalence, incidence, and determinants of major conditions of the elderly, including cerebrovascular disease (transient ischemic accident [TIA] and stroke), dementia, Parkinson disease, and essential tremor.^20–32^

Detailed accounts of the study population and sampling methods have been published.^20–32^ The survey area comprised 3 communities: Margaritas (approximately 14,800 inhabitants), which is a working-class neighborhood in Getafe (Greater Madrid); Lista (approximately 150,000 inhabitants), a professional-class neighborhood in the Salamanca district (Central Madrid); and Arévalo (approximately 9000 inhabitants), the agricultural zone of Arévalo County (125 km northwest of Madrid). Up-to-date lists of residents were generated from population registers. In each community, survey eligibility was restricted to residents aged 65 years or older who were present on December 31, 1993, or during 6 or more months of 1993. Eligible persons who had moved away from the survey area were not traced. In Margaritas and Arévalo, every eligible subject was selected for screening. However, due to the large number of elderly residents in Lista, proportionate stratified random sampling was used to select a subsample of 2113 subjects for screening. The selected study population was 6395 people, but 481 people were ineligible (census issues, address errors, or death), leaving 5914 eligible subjects, of whom 5278 were enrolled. All procedures were approved by the ethical standards committees on human experimentation at the University Hospitals “12 de Octubre” (Madrid) and “La Princesa” (Madrid). Written (signed) informed consent was obtained from all participants upon enrollment.

### Study Evaluation

Face-to-face evaluations were performed at baseline (1994–1995) and then at follow-up (1997–1998). Briefly, at the time of their baseline assessment, 5278 elderly subjects were interviewed face-to-face using a 500-item screening questionnaire during which data were collected on demographics, current medical conditions, smoking (ever vs never), and drinker (ever at least once per week vs never). As in prior studies,^18,33^ at baseline, subjects were asked to rate their current health on a 5-point scale using the question, “In general terms, how would you describe your health: very good, good, fair, poor, or very poor?” A small number of subjects were in several categories (eg, there were only 149 who described their health as very poor). Therefore, as suggested in several previous studies,^18,33^ we collapsed response options into 3 categories. These 3 categories were very good/good, fair, and poor/very poor.

A comorbidity index was calculated based on the presence of the following conditions, consistent with a recently published comorbidity score developed in ambulatory care settings:^34^ atrial fibrillation, cancer, chronic obstructive pulmonary disease, dementia, diabetes, epilepsy (treated), heart failure, myocardial infarction, depression, other psychiatric disorders (psychosis, schizophrenia, or bipolar affective disorder), renal disease, and stroke. The presence of some items resulted in the assignment of more points than others. The score ranged from 0 (no conditions) to 28 (ie, all conditions present).

A 37-item version of the Mini-Mental State Examination (37-MMSE) was also administered.^18,25,26,33,35^ The 37-MMSE was a Spanish adaptation of the standard MMSE. It included all of the standard MMSE items as well as 3 additional items: an attention task, that is, “say 1, 3, 5, 7, 9 backwards,” a visual order, that is, a man raising his arms, and a simple construction task, that is, copying 2 overlapping circles.^18,25,26,33,35^

Waist circumference was measured using a flexible nonelastic measuring tape. Individuals stood with feet together and arms resting by their sides.^36^ The waist circumference was taken as the narrowest point between the inferior rib border and the iliac crest.^36^

Most important for this study, the questionnaire included 4 screening questions for stroke and TIA:^23,24^ Have you ever been diagnosed by a physician as having suffered a stroke? Have you ever had slurred speech or problems talking to somebody? Have you ever felt mouth deviation? Have you ever felt weakness in an arm or a leg?

Participants who screened positive for cerebrovascular disease (N = 678) were examined by 1 of 8 senior neurologists who met at the inception of the study to establish standardized methods to perform and interpret the examination (JB-L, AM-S, and FB-P, and see http://www.ciberned.es/estudio-nedices: AB, JD-G, JO, JP, and JP-E).^23^ For 47 participants (6.9% of 678) who could not be examined, medical records were obtained from their general practitioners, from in-patient hospitalizations and from neurological specialists (if they had visited 1). We applied the World Health Organization clinical definition of a stroke: “rapidly developing clinical signs of focal (or global) disturbance of cerebral function lasting more than 24 hours (unless interrupted by surgery or death) with no apparent cause other than a vascular origin.”^23,24^ TIA was defined as a focal neurological deficit of sudden onset occurring in a cerebrovascular territory distribution and recovering without sequelae in less than 24 hours, with nonvascular causes excluded.^23,24^ Stroke and TIA classification and etiology was performed on the basis of medical records, reports of brain imaging, and the judgment of the NEDICES neurologists.^23,24^ We restricted the current analyses to those TIAs or strokes caused by symptomatic intracranial or extracranial atherosclerosis (hereafter called atherosclerotic stroke), including lacunar strokes. We included lacunar strokes in this category, since emerging evidence suggests that both large artery and small vessel disease should be considered together.^37^

With respect to coronary disease (ie, myocardial infarction or angina), the screening questionnaire included queries adapted and validated in Spanish from the Multinational MONItoring of trends and determinants in CArdiovascular disease and the Rose questionnaire on chest pain and dyspnea.^38^ The hospital records from those participants who screened positive for coronary disease were examined in order to confirm the cardiovascular event.^38^ The diagnosis of myocardial infarction was confirmed if data in the hospital record met standardized criteria of chest pain or equivalent, there were diagnostic electrocardiographic changes, and/or there were elevated cardiac enzyme levels.^38^ Angina was confirmed by hospital records.^38^

Participants were classified as having symptomatic atherosclerotic disease if they were diagnosed with noncardioembolic TIA or stroke, or coronary disease (angina or myocardial infarction).

### Exclusions and Final Sample for Analyses

**FIGURE 1:**
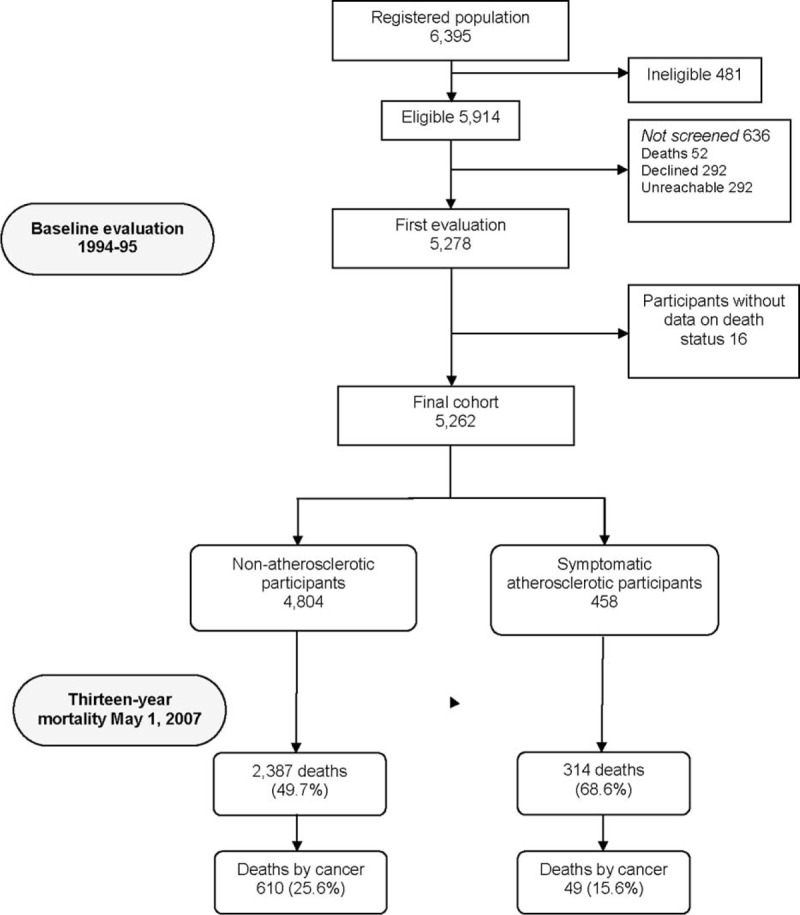
Flow chart of the study.

Beginning in January 1994, letters explaining the survey and inviting participation were mailed to 6395 subjects. Of these, 5914 subjects were deemed eligible for screening and 5278 subjects (89.2%) were screened. The 636 subjects who were not screened either declined (292, 45.9%), could not be located due to an address change (292, 45.9%), or had died (52, 8.2%) (Figure 1). Of the 5278 participants screened at the baseline evaluation, 16 were excluded because they lacked data on death status. Therefore, the final cohort consisted of 5262 participants.

Follow-up data on death were collected until May 1, 2007. The date of death was obtained from the National Population Register of Spain (Instituto Nacional de Estadística). In Spain, a doctor completes a death certificate on all individuals at the time of death. The certificate is then sent to the local authority in the municipality where the person had been living, and the information is collected in the National Register. The doctor completing the death certificate does so in accordance with the recommendations of the World Health Organization. The assignment of cause of death is based on the basic cause of death (http://www.who.int/topics/mortality/en/), defined as the illness or injury which started the chain of pathological events that directly led to death (http://www.who.int/topics/mortality/en/). Using the International Classification of Diseases (9th Revision for deaths that occurred prior to 1999, [http://www.cdc.gov/nchs/icd/icd9.htm] and 10th Revision [http://www.cdc.gov/nchs/icd/icd10.htm] for deaths occurring from 1999 to 2007), the NEDICES investigators classified the cause of death into 6 main categories: dementia, cerebrovascular disorders, cardiovascular disorders (pulmonary embolism, congestive heart failure, myocardial infarction, heart or aortic rupture, and asystole), respiratory diseases, cancer, and other causes (infections, trauma, genitourinary, or gastrointestinal disorders).

### Statistical Analyses

Data analyses were performed in SPSS Version 21.0 (IBM Corp., NY, USA). Age, waist circumference, 37-MMSE score, number of medications, and comorbidity index were not normally distributed (all Kolmogorov–Smirnov test, *P* < 0.001), even after log-transformation. Therefore, we used Mann–Whitney *U* test to analyze these continuous variables. The Chi-square test was used to analyze categorical variables.

We used Cox proportional hazards models to estimate the relative risk of cancer-specific mortality; this generated hazard ratios (HR) with 95% confidence intervals (CI). The time variable was the years from the date of the baseline evaluation (1994–1995) to either May 1, 2007 in living participants or the date of death in participants who had died prior to May 1, 2007.

We considered several baseline variables as potential confounders. These were age in years, gender, educational level, geographical area (Lista, Arévalo, and Las Margaritas), ever smoked (ex-smokers and current smokers), ever drank (ex-drinkers and current drinkers), waist circumference in cm, 37-MMSE total score, arterial hypertension, hypercholesterolemia, number of medications, comorbidity index, and self-rated health (good or very good, fair, and poor or very poor).

In Cox proportional hazards analyses, we estimated the risks of cancer-specific mortality in participants with symptomatic atherosclerotic disease compared with the reference group (participants who did not have symptomatic atherosclerotic disease). Then, in adjusted models, we first considered variables that were associated at the p < 0.05 level with both symptomatic atherosclerotic disease and cancer-specific mortality (“Model 1” [more restrictive criteria for confounding]) and then considered baseline variables that were associated at the p < 0.05 level with either symptomatic atherosclerotic disease or cancer-specific mortality (“Model 2” [less restrictive criteria for confounding]). For completeness, we also adjusted for all the potential confounders, independent of their statistical significance (ie, even if they were not associated with either the exposure or the outcome) (“Model 3”). In secondary analyses, participants with symptomatic atherosclerotic disease were stratified into 2 groups based on their smoking status.

Kaplan–Meier survival curves for subjects with and without symptomatic atherosclerotic disease were assessed; the log-rank test was used to compare the differences between the curves.

## RESULTS

The 5262 participants had a mean duration of follow-up of 9.5 years (median = 12.1 years; range = 0.01–13.5 years). Of the 5262 participants, 458 had suffered a symptomatic atherosclerotic disease event at baseline, including 201 TIA/stroke cases (43.9%), 235 (51.3%) angina/myocardial infarction cases, and 22 (4.8%) cases with both diseases.

A total of 2701 (53.5%) of 5262 participants died, with deaths occurring at a median follow-up of 6.2 years (range 0.01–13.3 years), including 314 (68.6%) deaths among 458 participants with symptomatic atherosclerotic disease and 2387 (49.7%) deaths among 4804 participants without atherosclerotic disease (Figure 1). There were significant differences in age, gender, smoking status, waist circumference, and medical comorbidities when participants with versus without symptomatic arteriosclerotic disease were compared (Table 1). In addition, participants with symptomatic atherosclerotic disease reported their health as poorer or much poorer when compared with participants without symptomatic arteriosclerotic disease.

**TABLE 1:**
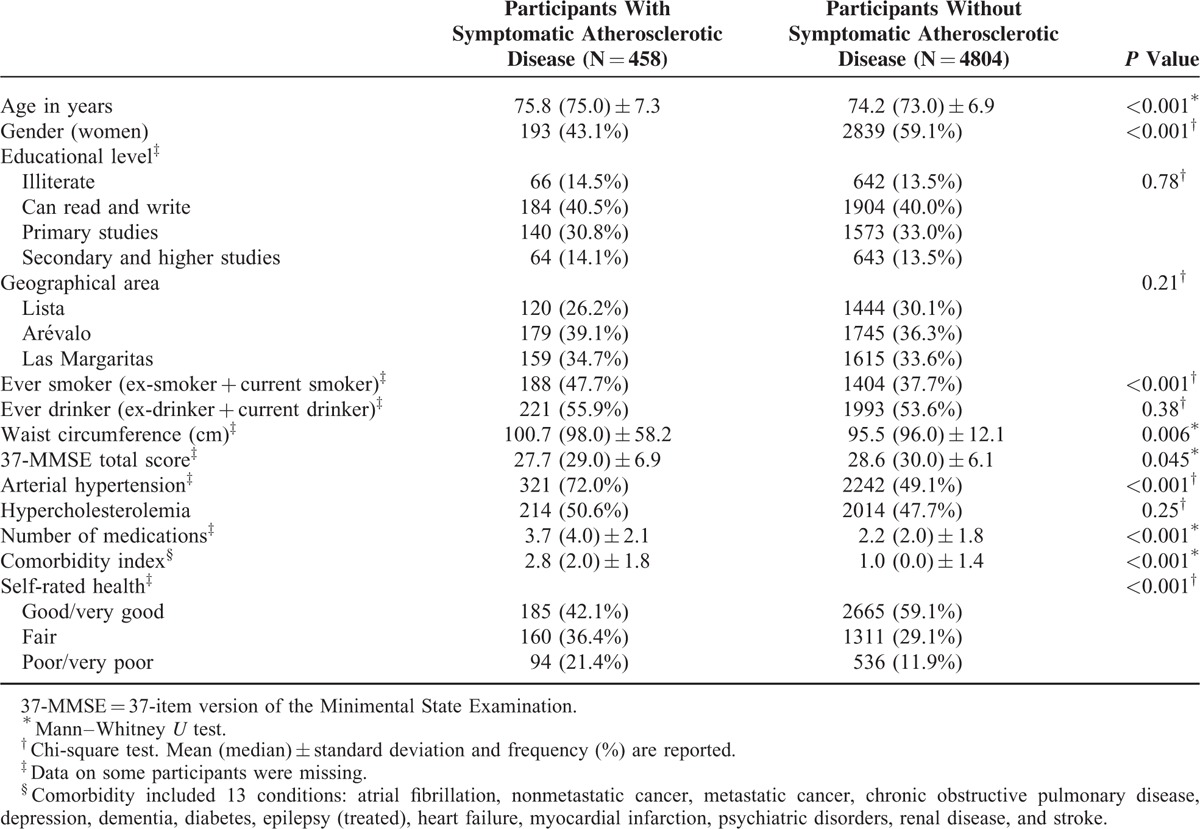
Baseline Demographic and Clinical Characteristics of Participants With Symptomatic Atherosclerotic Disease and Participants Without Symptomatic Atherosclerotic Disease

Baseline characteristics of the participants who died of cancer versus other causes are shown (Table 2). Those who died of cancer were more likely to be men and were younger and more educated. In addition, they were less likely to have hypertension and had a lower comorbidity index. They scored more highly on the 37-MMSE, and larger proportions were drinkers or smokers (Table 3). Further, they self-rated their health more highly than those who died of other causes (Table 3).

**TABLE 2:**
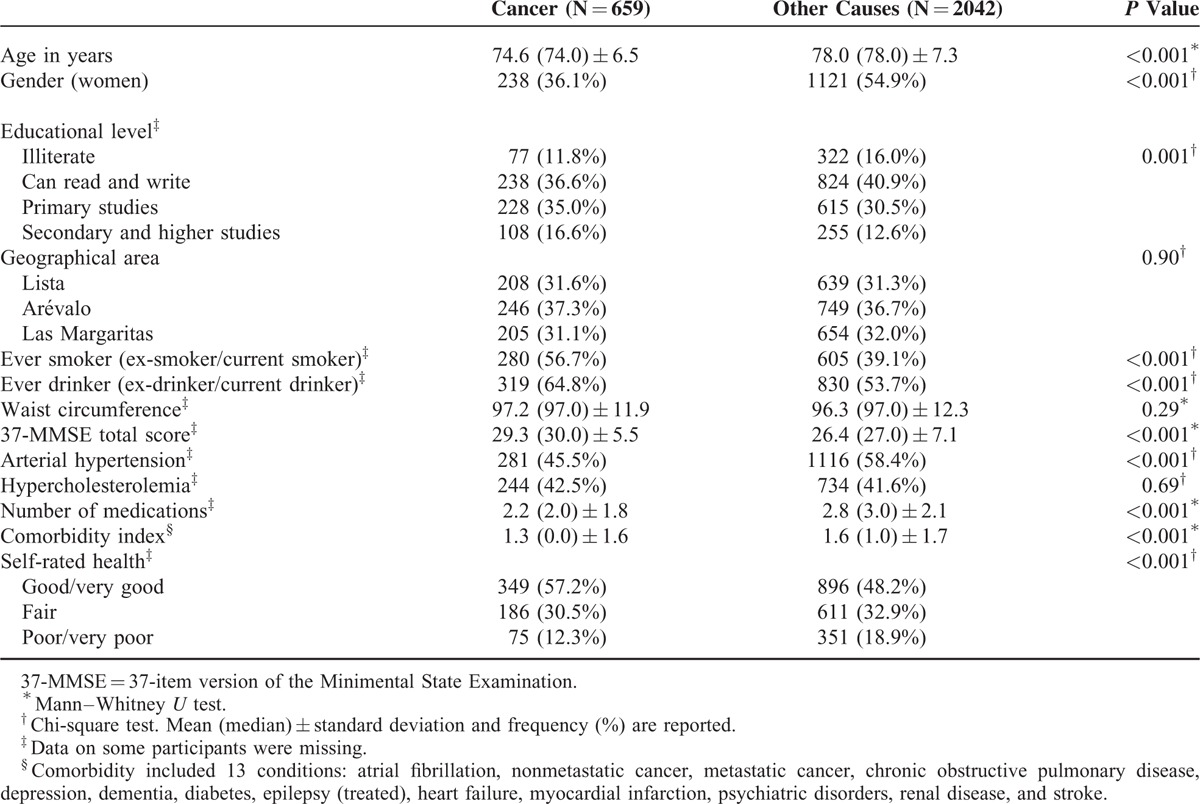
Baseline (1994–1995) Demographic and Clinical Characteristics of Participants Who Died of Cancer Versus Other Causes

**TABLE 3:**
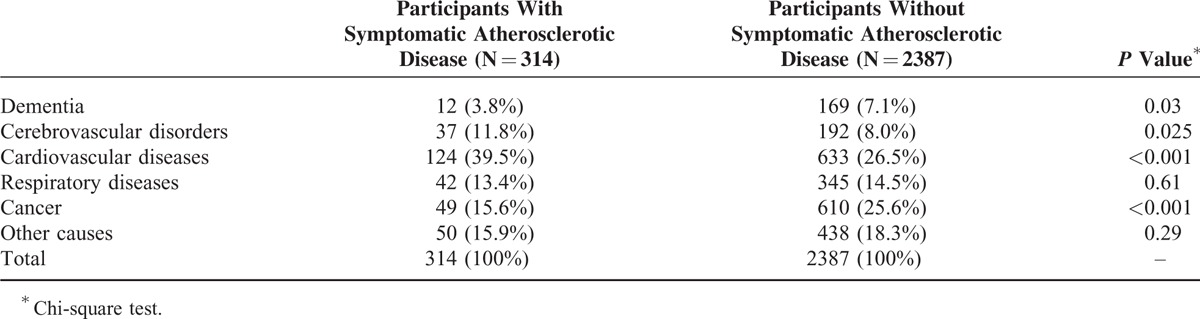
Cause of Death by Symptomatic Atherosclerotic Disease Status

Cause of death noted on the death certificates differed significantly by atherosclerotic disease status (Table 3). Cancer and dementia were reported significantly less often in participants with symptomatic atherosclerotic disease. By contrast, as expected, cerebrovascular and cardiovascular diseases were reported significantly more often in participants with symptomatic atherosclerotic disease (Table 3).

Type of cancer listed on the death certificate was compared in participants with versus without symptomatic atherosclerotic disease; malignant neoplasms of digestive organs and peritoneum were less frequent in participants with symptomatic atherosclerotic disease (Table 4).

**TABLE 4:**
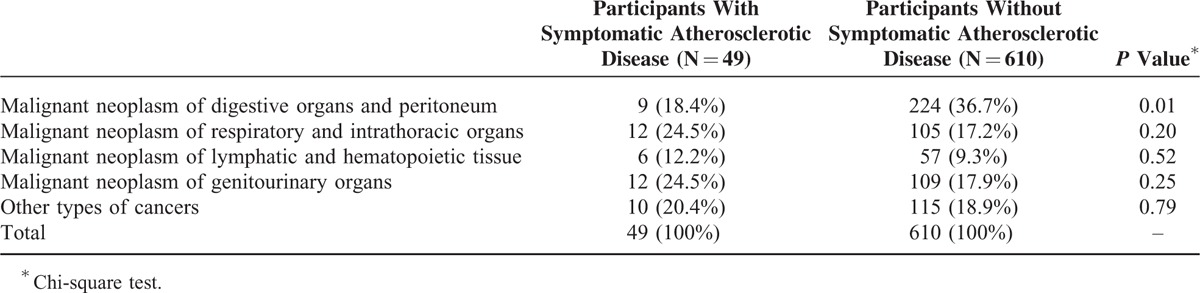
Types of Cancers (N = 659) Listed on the Death Certificate by Symptomatic Atherosclerotic Disease Status

In an unadjusted Cox model, risk of cancer-specific mortality was decreased in participants with symptomatic atherosclerotic disease (HR = 0.74, 95% CI, 0.55–0.98, *P* = 0.04) versus those without symptomatic atherosclerotic disease (reference group). After adjustment for baseline age, gender, ever smoker, 37-MMSE total score, arterial hypertension, number of medications, comorbidity index, and self-rated health (ie, variables that were associated with both symptomatic atherosclerotic disease and cancer-specific mortality), the risk of cancer-specific mortality remained decreased in participants with symptomatic atherosclerotic disease (HR = 0.67; 95% CI, 0.48–0.96, *P* = 0.03, Model 1 in Table 5). The results did not change in a Cox model that adjusted for variables that were associated with either symptomatic atherosclerotic disease or cancer-specific mortality (baseline age, gender, educational level, ever smoker, ever drinker, waist circumference, 37-MMSE total score, arterial hypertension, number of medications, comorbidity index, and self-rated health) (Model 2 in Table 5). Further, the results did not change in a Cox model that adjusted for all variables independent of their association with the exposure or outcome variables (Model 3 in Table 5).

**TABLE 5:**
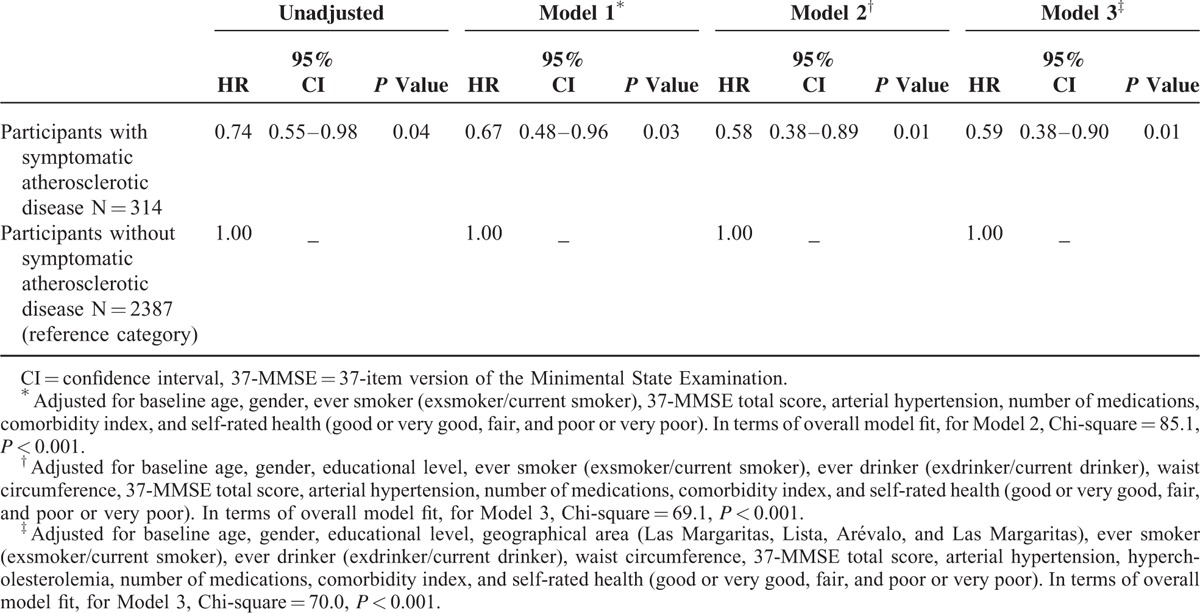
Risks of Cancer-Specific Mortality in Participants With Symptomatic Atherosclerotic Disease Versus Those Without Symptomatic Atherosclerotic Disease (Reference Group)

In a secondary analysis, participants with symptomatic atherosclerotic disease were stratified into 2 groups based on their smoking status. In nonsmokers, the risk of cancer-specific mortality was decreased in participants with atherosclerotic disease (unadjusted HR = 0.48; 95% CI, 0.25–0.94, *P* = 0.03). In adjusted analyses, for nonsmokers, the results remained similar (HR = 0.48; 95% CI, 0.24–0.97, *P* = 0.04, Model 1; HR = 0.33; 95% CI, 0.12–0.93, *P* = 0.04, Model 2; and HR = 0.35; 95% CI, 0.12–0.98, *P* = 0.04, Model 3). In ever smokers, the risk of cancer-specific mortality was similar in participants with versus without symptomatic atherosclerotic disease (unadjusted HR = 0.78; 95% CI, 0.54–1.13, *P* = 0.19).

In an additional secondary analysis, participants with symptomatic atherosclerotic disease were stratified by gender. In men, the risk of cancer-specific mortality was decreased in participants with atherosclerotic disease (unadjusted HR = 0.65; 95% CI, 0.47–0.91, *P* = 0.01). In adjusted analyses, for men, the results remained similar (HR = 0.64; 95% CI, 0.43–0.96, *P* = 0.03, Model 1; HR = 0.63; 95% CI, 0.39–1.0, *P* = 0.05, Model 2; and HR = 0.62; 95% CI, 0.38–1.0, *P* = 0.05, Model 3). In women, the risk of cancer-specific mortality was lower, but not to a significant degree, in participants with versus without symptomatic atherosclerotic disease (unadjusted HR = 0.68; 95% CI, 0.38–1.22, *P* = 0.20).

In a final analysis, we excluded gender-specific cancers such as breast cancer (N = 25) and prostate cancer (N = 54). In an unadjusted Cox model, risk of cancer-specific mortality was decreased in participants with symptomatic atherosclerotic disease (HR = 0.74, 95% CI, 0.55–0.98, *P* = 0.04) versus those without symptomatic atherosclerotic disease (reference group). In adjusted analyses, the results remained similar (HR = 0.72; 95% CI, 0.50–1.04, *P* = 0.08, Model 1; HR = 0.62; 95% CI, 0.40–0.98, *P* = 0.04, Model 2; and HR = 0.63; 95% CI, 0.40–0.99, *P* = 0.04, Model 3).

**FIGURE 2:**
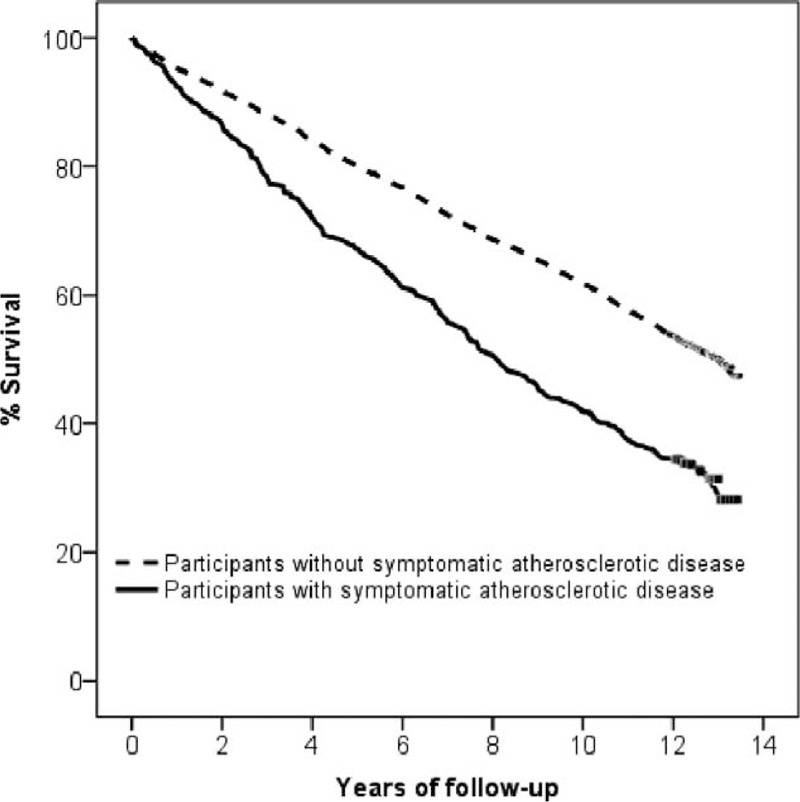
Kaplan–Meier survival curves for subjects with and without symptomatic atherosclerotic disease. The former had an increased risk of death (log-rank *P* < 0.001).

The Kaplan–Meier curve for overall survival (Figure 2) showed the atherosclerotic cohort to be at increased risk of death (log-rank *P* < 0.001).

## DISCUSSION

The current study suggests that people with symptomatic atherosclerotic disease are at reduced risk of mortality from malignant neoplasm. When compared to participants without symptomatic atherosclerotic disease, the risk for neoplasm as the underlying cause of death was approximately 30% to 40% lower in participants who had symptomatic atherosclerotic disease. Results from our prospective long-term follow-up population-based study support earlier results from a clinico-pathological study of an inverse correlation between atherosclerosis and cancer.^39^

A limited number of studies on the incidence of cancer among those who suffered from cerebrovascular or cardiovascular disease have been conducted, and the results are inconclusive.^12–15^ In a retrospective cohort of nearly 70,000 patients discharged with extra-coronary manifestations of atherosclerosis, from the Danish National Registry of Patients, Dreyer and Olsen^13^ found no association between atherosclerosis and colorectal cancers or hormone-related cancers, except for a decreased standardized incidence ratio of 0.7; 95% CI, 0.5 to 0.9 for endometrial cancer. Again, using the same cohort, these authors found that the rates of colorectal cancer in acute myocardial infarct patients were similar to those of the general population, as were the rates for hormone-related cancers, including endometrial and postmenopausal breast cancers.^12^ However, they found a moderate increase in the risk for tobacco-related cancers, which was strongest for patients with early onset of acute myocardial infarct and for female patients.^12^ In the same cohort, atherosclerotic women had a significantly elevated HR for lung cancer (3.26; 95% CI, 1.95–5.46).^14^ Finally, in the Danish nationwide cohort, patients with acute myocardial infarction and stroke were at 85% and 42% increased risk of being diagnosed with colorectal cancer, respectively, within the first year of diagnosis.^15^ However, no increased risk in the second and subsequent years was found.^15^ Our results extend prior knowledge for the overall finding of lower risk of cancer-specific mortality in the setting of symptomatic atherosclerotic disease. The fact that the risk of cancer mortality was significantly lower in nonsmokers with atherosclerotic disease, but not in ever-smokers with atherosclerotic disease, suggests that a genetic or epigenetic predisposition may mediate this inverse association.

Although the current findings suggest that symptomatic atherosclerotic disease is associated with decreased cancer-specific mortality, the mechanisms underlying this association remain unknown. One of the specific links between atherosclerosis and cancer could be the sirtuin family of proteins (SIRT1–7).^40^ Sirtuin functions are intrinsically linked to cellular metabolism, lifespan extension induced by caloric restriction, and gene expression.^41,42^ It has been shown that SIRT1 is a critical protective gene in the vasculature and that decreased SIRT1 expression may promote atherosclerosis and medial degeneration due to DNA damage, apoptosis, and accelerated cell ageing.^41,42^ Alzheimer disease, as well as other neurodegenerative diseases, has been associated with a reduced risk of cancer mortality,^17^ and down regulation of the sirtuin pathway has been shown to play a crucial role in the pathogenesis of Alzheimer disease.^43^ Conversely, overexpression of SIRT1 is frequently observed in various types of cancers.^44^ Although it remains a controversial issue, it seems likely that SIRT1 overexpression confers survival advantages to cancerous or transformed cells and accelerates carcinogenesis.^44^

Further, elevated levels of β-catenin protein, a hallmark of an activated canonical Wnt pathway, have been demonstrated in common forms of human malignancies, creating a favorable microenvironment for tumor growth and metastasis and contributing to therapeutic resistance.^45^ Conversely, patients with atherosclerotic vascular disease had significantly higher levels of Dickkopf-1, a potent inhibitor of Wnt signaling.^46^ Other crucial and evolutionarily conserved signaling pathways, such as p53, p21, and p16, are significantly upregulated in atherosclerotic disease,^47,48^ while they are frequently inactivated in human tumors.^49^

Our study has limitations. First, we did not collect data on comorbidity at death or data on who signed the death certificate (general physician vs neurologist vs oncologist or geriatrician). It is logical to assume that the level of expertise of the physician who signed the death certificate would predict the level of accuracy of that certificate. Second, we based the diagnoses of cancer on death certificates. The quality of cancer death certification in Spain is considered high.^50^ Third, NEDICES was a study of the elderly; hence, we only included individuals aged 65 and older. This means that our findings may not necessarily be generalized to younger populations. However, the prevalence and incidence of both cancer and symptomatic atherosclerotic disease is highest in the age group we enrolled,^1–3^ with the disease burden being of most importance in older age groups. Fourth, it is possible that risk factors could have changed during the interim follow-period. Fifth, residual confounding by unmeasured variables in Cox proportional hazards analyses is possible. Finally, competing mortality is an issue to consider – indeed, there could be a potential for survival bias, in that a small proportion of individuals may have died of cancer at younger ages. However, as we stated previously, both cancer and atherosclerotic disease are ageing-related diseases. Therefore, we do not feel that competing mortality could have biased our results.

This study also had several strengths. First, the design was population-based, allowing us to assess people with symptomatic atherosclerotic disease not based solely on care-seeking behavior and, therefore, the results may be generalizable to the community. Second, the assessments were conducted prospectively in a standardized manner, and the follow-up was of considerable length. Third, we were able to adjust for the potential confounding effects of a number of important factors. Fourth, we had the ability to consider detailed analyses of smoking status. Finally, we performed a detailed clinician and chart based-assessment of symptomatic atherosclerosis.

Using a prospective, population-based design, we demonstrated that symptomatic atherosclerotic disease was associated with decreased risk of cancer-specific mortality. Of note was that the risk of cancer mortality among those who had symptomatic atherosclerosis, but had ever smoked, was similar to the general population. It is as if tobacco were a modulator of the overall risk. This study provides evidence of an inverse association between symptomatic atherosclerotic disease and cancer.
